# A Reformed PSO-Based High Linear Optimized Up-Conversion Mixer for Radar Application

**DOI:** 10.3390/s24030879

**Published:** 2024-01-29

**Authors:** Tahesin Samira Delwar, Unal Aras, Abrar Siddique, Yangwon Lee, Jee-Youl Ryu

**Affiliations:** 1 Department of Smart Robot Convergence and Application Engineering, Pukyong National University, Busan 48513, Republic of Korea; samira.fset@gmail.com (T.S.D.); unalaras21.20@gmail.com (U.A.); 2 Department of Global IT Engineering, Kyungsung University, Busan 48434, Republic of Korea; abrarkhokhar.iiui@gmail.com; 3 Department of Spatial Information Engineering, Pukyong National University, Busan 48513, Republic of Korea

**Keywords:** enhanced cross-quad transconductor, reformed particle swarm optimization, radar, two-fold transconductance path, up-conversion mixer

## Abstract

A reformed particle swarm optimization (R_PSO_)-based up-conversion mixer circuit is proposed for radar application in this paper. In practice, a non-optimized up-conversion mixer suffers from high power consumption, poor linearity, and conversion gain. Therefore, the R_PSO_ algorithm is proposed to optimize the up-conversion mixer. The novelty of the proposed R_PSO_ algorithm is it helps to solve the problem of local optima and premature convergence in traditional particle swarm optimization (T_PSO_). Furthermore, in the R_PSO_, a velocity position-based convergence (VP_C_) and wavelet mutation (W_M_) strategy are used to enhance R_PSO_’s swarm diversity. Moreover, this work also features novel circuit configurations based on the two-fold transconductance path (T_TP_), a technique used to improve linearity. A differential common source (D_CS_) amplifier is included in the primary transconductance path (P_TP_) of the T_TP_. As for the subsidiary transconductance path (S_TP_), the enhanced cross-quad transconductor (E_CQT_) is implemented within the T_TP_. A benchmark function verification is conducted to demonstrate the effectiveness of the R_PSO_ algorithm. The proposed R_PSO_ has also been compared with other optimization algorithms such as the genetic algorithm (GA) and the non-dominated sorting genetic algorithm II (NSGA-II). By using R_PSO_, the proposed optimized mixer achieves a conversion gain (CG) of 2.5 dB (measured). In this study, the proposed mixer achieves a 1 dB compression point (OP_1_dB) of 4.2 dBm with a high linearity. In the proposed mixer, the noise figure (NF) is approximately 3.1 dB. While the power dissipation of the optimized mixer is 3.24 mW. Additionally, the average time for R_PSO_ to design an up-conversion mixer is 4.535 s. Simulation and measured results demonstrate the excellent performance of the R_PSO_ optimized up-conversion mixer.

## 1. Introduction

**Figure 1:**
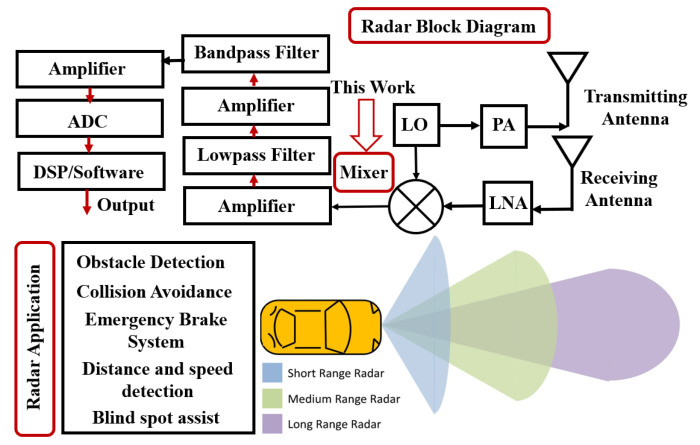
A 24 GHz overview (block diagram and radar application).

In recent years, radar technology has evolved significantly in many areas such as automotive safety, aerospace, and industrial automation [1]. A typical automotive radar operates at a millimeter-wave frequency near 24 or 77 GHz [2]. As an overview, Figure 1 shows the radar system in a nutshell. As seen in the block diagram below, a transceiver module, an antenna, a signal processing unit, and a control interface are the four main components of a typical radar system operating at 24 GHz. In automotive applications, a 24 GHz radar can be employed for adaptive cruise control, collision avoidance, and blind spot detection [3].

One of the critical components of a radar system is the mixer, which plays a significant role in signal processing and up-conversion from the RF (Radio Frequency) to the IF (Intermediate Frequency) stage. A radar system requires frequency translation before subsequent processing and analysis. The high-performance mixer in radar systems can detect objects at a wide range of distances and speeds [4].

However, poor linearity results in inadvertently producing signals outside of the spectrum allocated to radar applications, which can result in interference with other communication systems [5]. This is why it is imperative that communication systems and radars adhere to strict linearity standards so that harmonious coexistence within the dedicated frequency bands can be maintained. For this reason, radar applications require high linearity. High linearity of the mixer ensures that radar signals’ modulation characteristics are maintained accurately. Moreover, the linearity and power efficiency trade-off is often one of the major drawbacks of traditional mixer design [6]. On the other hand, mixers also suffer from compromised linearity if they attempt to minimize power consumption, limiting their ability to handle interfering signals and weak target echo signals. As a consequence, it has become important to optimize up-conversion mixers geared toward 24 GHz applications.

Specifically, this paper addresses the challenges of linearity in up-conversion mixer design at 24 GHz. A reformed particle swarm optimization (R_PSO_) is proposed to design a two-fold transconductance path (T_TP_)-based up-conversion mixer to achieve high linearity. The advantage of R_PSO_ is it excels at balancing global exploration and local exploitation. Also, in the designed up conversion mixer, the R_PSO_’s enhanced convergence speed, which allows quick identification of optimal configurations. In mixer design, where multiple parameters must be optimized simultaneously, the algorithm’s ability to handle a large solution spaces is helpful.

### 1.1. Related Literature

In the early 1980s, Eberhart and Kennedy presented an algorithm for optimizing continuous nonlinear functions using the concept of traditional particle swarm optimization (T_PSO_), which relies on the collective behavior of social swarms [7]. In recent years, T_PSO_ has become one of the most powerful optimization tools due to the fact that each particle represents a feasible solution in the solution space.

However, in practise, it is hard to control circuit configurations accurately with commercial CAD tools. The design process requires a highly skilled individual, and it can be quite time-consuming and complicated [8]. In such cases, optimization algorithms are required. It is common to see a lot of discussion in the literature regarding genetic algorithms (GAs), simulated annealing (SA), firefly algorithms (FAs), and particle swarm algorithms (PSOs), all of which are effective when implemented on analog circuits, such as power amplifier (PA), operational amplifier (Op-amp), voltage controlled oscillators (VCO), and, low noise amplifier (LNA) [2]. In spite of this, a mixer block optimization has not been studied very widely. A mixer design can be optimized through the exploration of multidimensional parameter spaces and the computation of optimal solutions. As part of our work, we demonstrate how R_PSO_ can be implemented on a mixer circuit. In an integrated RF circuit design process, R_PSO_ has a significant impact because it is an effective and quick method of optimizing complex circuit parameters, reducing the length of the design cycle, enhancing performance, and addressing various differences which affect the performance of RF circuits.

In this paper, Mugadhanam et al. [9] describe a three-stage low noise amplifier (LNA) based on the cascode technique. The proposed work addresses several shortcomings of prior LNAs, including a minimal noise figure (NF), high linearity, and low power dissipation. However, this work may need to conduct further testing to determine whether the design is scalable, robust, and compatible with other technologies, frequencies, or systems. Inverter cascode (InvCas) is a transimpedance amplifier (TIA) for optical receivers that is presented by Elbadry et al. [10]. The main objective of this paper is to identify the necessary circuit parameters needed to obtain the target specifications by using particle swarm optimization (PSO) combined with the gm/ID methodology. Even though the paper focuses on the design of the InvCas TIA for the optical receiver’s front end, the proposed work is not discussed in any further detail. According to Zhao et al. [11], PSO algorithms are combined with AI-integrated neural networks to create a model for semiconductor optical amplifiers (SOAs). Based on the curve of the tested SOA performance, this model enhances the effectiveness of SOA design. This paper [12], uses the firefly algorithm (FA), particle swarm optimization (PSO), and genetic algorithm (GA) to optimize variable gain LNA (VG-LNA). In VG-LNA, a complementary common gate (CCG) is coupled with a VGA. In comparison to GA and PSO, FA generates better results than all three optimization techniques. A Doherty amplifier is presented in this paper, and a low and a high power region are analyzed [13]. The paper also expands the application of the automatic PA design. However, neither a multi-objective optimization strategy for the proposed automatic PA design system nor its specific limitations are discussed in this paper. A genetic algorithm (GA) is used in this study to optimize two Bulk CMOS technology nodes of low noise amplifiers (LNAs) [14]. Optimization of the LNA was significantly faster using the interactive optimization tool than using the non-interactive method.

A novel adaptive genetic algorithm (AGA) [15] was applied in this paper that enhances the circuit power-added efficiency (PAE) and solves trapping in local optimum. The proposed optimized PA design is not addressed in terms of its applicability to different frequency bands or to 5G. An optimized balun is achieved with a genetic algorithm by designing and implementing a wideband double-balanced mixer based on a GaAs pHEMT process [16]. This paper examines the use of artificial intelligence to optimize and improve the performance of analog and mixed-signal circuits [17]. A major challenge in AMS circuit automation is the reliance on circuit knowledge expertise, which can limit the effectiveness of AI-based approaches. Although mathematical optimizations have their advantages, they also carry some drawbacks, such as not being able to capture all the information contained in a complex physical model. In addition to circuit knowledge, AI techniques could be used to improve results. In terms of its computational efficiency, FA is superior to other methods such as particle swarm optimization (PSO), the cuckoo search algorithm (CSA), human behavior optimization (HB-PSO), in terms of simulations, comparative studies, and statistical analyses. In [18], using the current mirror technique, a double-balanced downconversion mixer is designed. Also, to optimize the circuit genetic algorithm (GA), the inclined plane system optimization (IPO) algorithm, and particle swarm optimization (PSO) have been used. As well as IPO, PSO and GA optimization algorithms have been used to optimize circuits. However, this work does not analyze computational efficiency in its entirety, and the proposed method might not be feasible for certain real-time applications.

This paper presents a novel R_PSO_-based optimization approach that has shown promising results. To maximize linearity and minimize power consumption, we optimized a 24 GHz T_TP_-based up-conversion mixer using R_PSO_. By integrating R_PSO_ into the mixer design, a more efficient and reliable radar system is designed.

In this paper, four sections are presented. T_PSO_ and R_PSO_ are discussed in Section 2. Also, Section 2 includes a proposed design for an optimized up-conversion mixer based on R_PSO_. The results and analysis are presented in Section 3. In Section 4, we draw the final section.

### 1.2. Main Contribution

1. In this paper, we present a novel algorithm, reformed particle swarm optimization (R_PSO_), which is designed for the optimization of the up-conversion mixer.
2. In particular, R_PSO_ is designed to avoid the problems of local optima and premature convergence associated with traditional particle swarm optimization (T_PSO_). Thus, this paper enhances the ability to explore a wider solution space and find better design configurations.
3. We introduce two novel R_PSO_ strategies to improve diversity: velocity position-based convergence (VP_C_) and wavelet mutation (W_M_). In this way, the proposed algorithm explores a broader range of solutions, resulting in a more robust and diverse search process.
4. The core concept behind the design of the optimized up-conversion mixer is the two-fold transconductance path (T_TP_). The objective of this proposed circuit is to increase the mixer’s linearity, which is a crucial feature for radar applications. This work achieves a high linearity and a high conversion gain through the proposed algorithm.

## 2. Research Methodolgy

**Figure 2:**
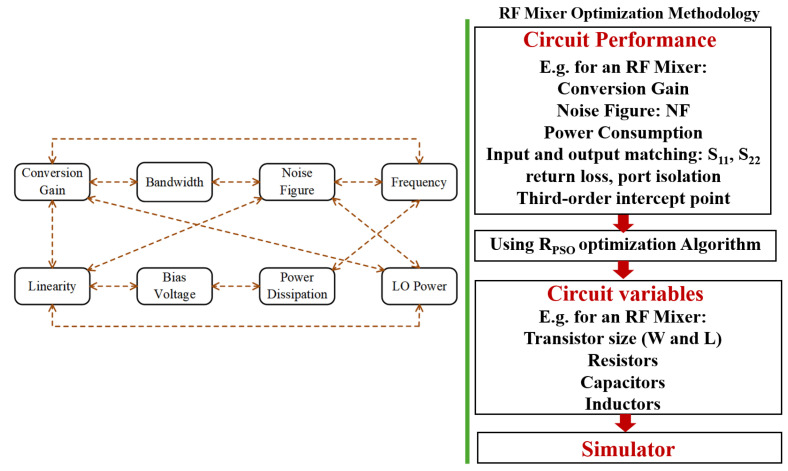
Overall flow chart of research methodology.

The research methodology focuses on the use of R_PSO_, which is adapted to meet specific challenges posed by up-conversion mixer challenges, to achieve high linearity. This study is likely to include simulation and experimentation phases to validate the methodology proposed in the study. Figure 2 depicts the overall flow chart of the research methodology.

### 2.1. Traditional PSO (T_PSO_)

A popular and effective optimization technique based on the social behavior of birds and fish is known as T_PSO_ [19].

**Figure 3:**
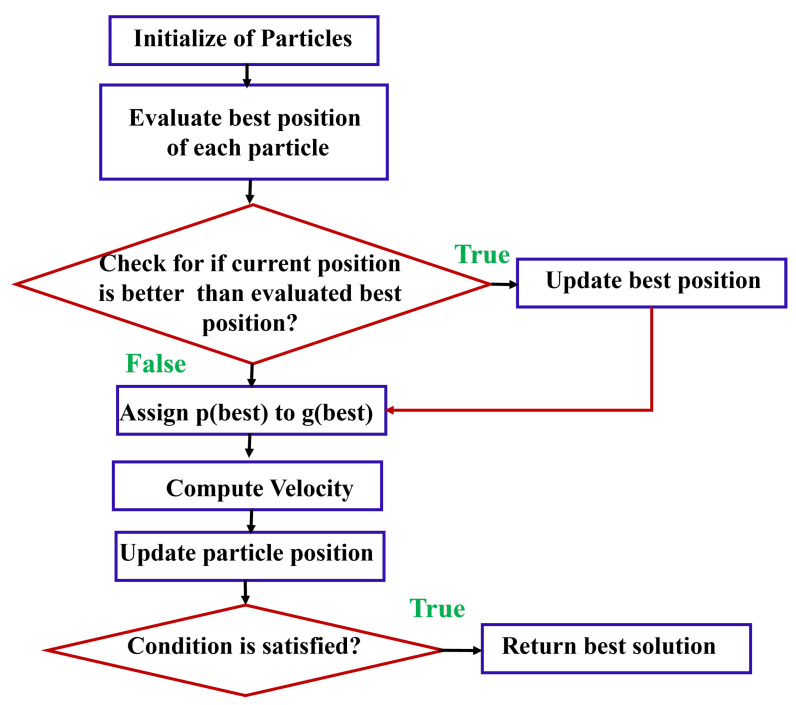
T_PSO_ algorithm.

1. A particle population is initialized in T_PSO_, each representing a potential solution. The particles are assigned random positions and velocities in the search space.
2. In the T_PSO_ process, we need to define each particle’s best-known position (individual best or p_best_) as its initial position, and the global best-known position g_best_ as the best position among all particles in the swarm.
3. Moreover, the next phase involves evaluating the particle’s current fitness in accordance with the objective function of the optimization problem, and comparing the particle’s current fitness to its best-known fitness p_best_.
4. The position should be updated if it is better than the current one.
5. Then compare each particle’s p_best_ to its g_best_. Whenever a particle’s p_best_ is better than a particle’s current g_best_, update g_best_ accordingly. The algorithm of T_PSO_ can be seen in Figure 3.

### 2.2. Proposed Reformed PSO (R_PSO_)

**Figure 4:**
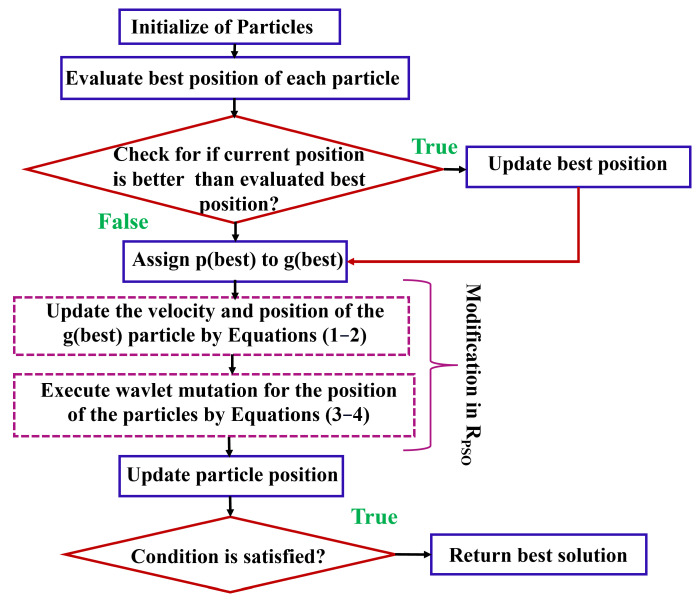
Proposed R_PSO_ algorithm.

In this work, the R_PSO_ refers to a modification of the T_PSO_ algorithm [20]. The R_PSO_ aims to enhance the efficiency, convergence speed, robustness, and performance of the proposed mixer circuit. In the proposed R_PSO_, the choice of modification depends on the strategy of velocity position-based convergence (VP_C_) and wavelet mutation (W_M_). In the below, we describe the strategy of VP_C_ and W_M_. Figure 4 shows the proposed R_PSO_ algorithm.

### 2.3. Velocity Position Based Convergence (VP_C_) Strategy

The VP_C_ is a modification introduced in R_PSO_ to enhance its convergence speed and solution quality. The VP_C_ adapts particle velocity and position updates to their historical information and swarm-wide best solutions. There is a set of best-known solutions maintained by the algorithm for each particle, referred to as the g_best_ and p_best_. A combination of historical and current data is used to determine velocity and position updates. The g_best_ position represents the best solution found by any particle in the entire swarm. Particles are attracted towards this position to exploit promising regions in the search space. Until now, each particle has maintained its p_best_ position. This encourages particles to explore regions that have been successful for them individually.

The VP_C_ strategy combines the exploration ability of the g_best_ position and the exploitation ability of each particle’s p_best_ position, promoting a balanced exploration–exploitation trade-off. The flexibility of adjusting the velocity based on both global and personal information contributes to the algorithm’s ability to converge efficiently towards optimal solutions in the search space.

The VP_C_ can be expressed as from Equations (1)–(3):

(1) $$u_{\xi d}\left(t+1\right)=-z_{\xi d}\left(t\right)+q_{gd}\left(t\right)+\omega u_{\xi d}\left(t\right)+\rho \left(t\right)\left(1-2_{j_{2d}}\left(t\right)\right)$$

(2) $$z_{\xi d}\left(t+1\right)=z_{\xi d}\left(t\right)+u_{gd}\left(t+1\right)=p_{gd}\left(t\right)+\omega u_{\xi d}\left(t\right)+\rho \left(t\right)\left(1-2_{j_{2d}}\left(t\right)\right).$$

Here, ξ = index of the g_best_ particle;

zξd(t) = Optimizes the position of the particle globally P_gd_(t);

ωxξd(t) = a present search;

$\rho \left(t\right)(1-2j_{2d}\left(t\right))$= samples array 2ρ(t);

P = a scaling factor designated below determines the g_best_ position.

(3) $$\rho \left(t+1\right)=\left\{\begin{array}{ccc} 2\rho \left(t\right), & if∼success>S_{c} & \\ 0.5\rho \left(t\right), & if∼failures>f_{c} & \\ p(t), & otherwise & , \end{array}\right.$$

*S_c_*, *f_c_* = denotes a current threshold.

There are consecutive successes and failures determined by the terms ∼ successes and ∼ failures. The failure here is defined as $f\left(p_{g}\left(t\right)\right)=f{\left(p\right)}_{g}\left(t-1\right))$, while the success is the exact opposite.

The VP_C_ strategy combines the exploration ability of the g_best_ position and the exploitation ability of each particle’s g_best_ position, promoting a balanced exploration–exploitation trade-off. The flexibility of adjusting the velocity based on both global and personal information contributes to the algorithm’s ability to efficiently converge toward optimal solutions in the search space.

### 2.4. Wavelet Mutation (W_M_) Strategy

The W_M_ strategy is an innovative technique employed in R_PSO_ to enhance the exploration capability of the algorithm. The goal is to provide an efficient method for exploring a variety of different regions within the search space through the integration of wavelet transformation principles into the mutation process of R_PSO_.

Based on the historical information in R_PSO_ that particles evolve by adjusting their p_best_ and g_best_ positions and velocities. There is no doubt that R_PSO_ is highly effective for optimization. However, by incorporating a mutation strategy into the search process, the whole process will be diversified and will be able to avoid becoming stuck in local optima. The W_M_ strategy introduces wavelet transformations to generate diverse perturbations in the search space. Wavelet transformations are mathematical operations that decompose a function into different frequency components, allowing for both global and local information extraction.

In the W_M_, mutation probability determines whether an individual particle will mutate; $P_{m}$ϵ [0, 1]. An individual particle position generates a random number (in between 0 to 1). Therefore, to be specific, $x_{i}\left(t\right)=\left[x_{i1}\left(t\right),x_{i2}\left(t\right),x_{ij}\left(t\right),x_{iD}\left(t\right)\right]$ consider a present selected particle, where $x_{ij}\left(t\right)$ = is the position of the j^th^ dimension in the iteration i^th^.

(4) $$\bar{x_{ij}}\left(t\right)=\left\{\begin{array}{cc} x_{ij}\left(t\right)+\sigma \times \left(P_{jmax}-x_{ij}\left(t\right)\right), & if\sigma >0\\ x_{ij}\left(t\right)+\sigma \times \left(x_{ij}\left(t\right)-p_{jmin}\right), & if\sigma \leq 0 \end{array}\right.$$

(5) $$\sigma =\frac{1}{\sqrt{a}}e\left(\frac{\varphi }{a}\right)\frac{2}{2}cos\left(5\times \left(\frac{\varphi }{a}\right)\right).$$

### 2.5. Proposed R_PSO_-Based Optimized Up-Conversion Mixer

In the context of RF electronics, a Gilbert mixer is a fundamental building block used in various applications, particularly in frequency conversion processes [21]. Gilbert mixers often suffer from nonlinearity, which means that the output signal contains unwanted harmonics and intermodulation products. This nonlinearity can lead to signal distortion and degradation in receiver performance. Also, some traditional mixers may suffer from LO leakage, where a portion of the LO signal leaks into the RF or IF path, causing unwanted interference and degradation of the desired signal. To address these issues, prior research has developed various techniques and improved mixer architectures to enhance linearity.

### 2.6. Circuit Design Explanation

**Figure 5:**
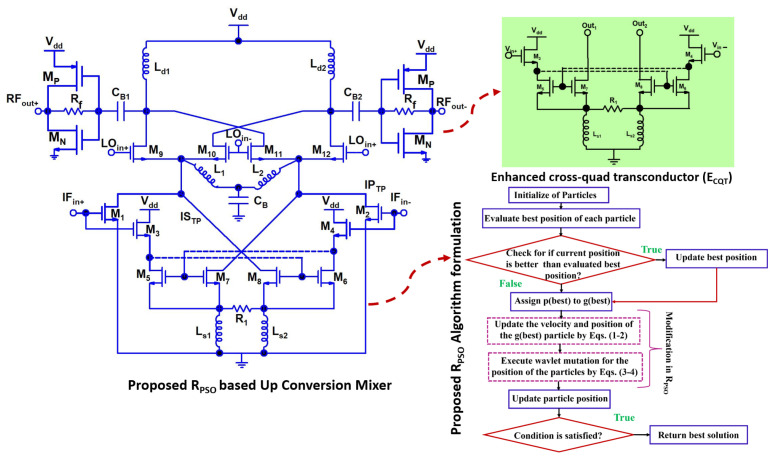
Schematic of a proposed optimized up-conversion mixer.

This work proposes an up-conversion mixer design based on R_PSO_. Figure 5 shows its schematic. The design mixer includes the enhanced cross-quad transconductor (E_CQT_) and differential common source (D_CS_) amplifier to amplify the input IF signal in the two-fold transconductance path (T_TP_). D_CS_ amplifiers are implemented by transistors M_1_, M_2_. In addition to R_7_ and R_8_, transistors M_7_ and M_8_ relate to a feedback resistor R_1_. The IF is applied at M_3_ and M_4_’s gate nodes in E_CQT_. With cross-coupling transistors M_5_–M_7_ and M_6_–M_8_, transistors M_3_ and M_4_ form a current mirror. To increase the mixer’s gain, the inductors L_1_, L_2_, and C_3_ are connected to the common nodes of the transconductance and switching stages. The transistors M_9_–M_12_ are connected to the gate nodes of transistors in the switching stage to receive a differential LO signal of 21.6 GHz. The switching stage translated the input of T_TP_ into a 24 GHz differential RF signal. A 50 Ohm output match is achieved by complementary MOS transistors M_n_ and M_p_ and resistor Rf, which act as the load for the RF stage. n/pMOS transistors M_n_ and M_p_ serve as the push–pull output buffer.

The primary transconductance path (P_TP_) and subsidiary transconductance path (S_TP_) are the two transconductance paths in the T_TP_ stage of a designed mixer. In the P_TP_, a pair of CS amplifiers operates in the saturation region, whereas the S_TP_ is composed of an E_CQT_ amplifier. The E_CQT_ is implemented in S_TP_ to improve linearity and transconductance. We have incorporated an E_CQT_-based S_TP_ into the designed mixer due to the linearity being the main concern at a frequency of 24 GHz, whereas only a P_TP_-based transconductance is not sufficient for radar applications. The E_CQT_ was chosen to enhance the g_m_ in the designed mixer. Positive feedback is avoided in E_CQT_ by using current mirror transistors M_5_, M_7_, and M_6_–M_8_ at the drain terminals, while linear output signals are taken at the source terminals. In our work, to optimize the up-conversion mixer using R_PSO_, the cost function we use is:

(6) $$CG=10\cdot log_{10}\left(\frac{P_{RF,out}}{P_{LO,in}}\right)$$

(7) $$P1dB_{out}=P1dB_{input}+\left(Gain-1\right)dBm.$$

### 2.7. Validation of R_PSO_ Using Benchmark Functions

This paper evaluates the effectiveness of R_PSO_ algorithms by conducting a benchmark function verification experiment. In this study, R_PSO_ and T_PSO_ are compared and analyzed. The simulations are all implemented in MATLAB R2023a. Equations (8)–(13) represent the benchmark functions for R_PSO_ validation.

(8) $$F_{1}=\sum_{i=1}^{n}x_{i}^{2}$$

(9) $$F_{2}=\sum_{i=1}^{n}{\left(\left[x_{i}+0.5\right]\right)}^{2}$$

(10) $$F_{3}=\sum_{i=1}^{n}ix_{i}^{4}+random\left[0.1\right]$$

(11) $$F_{4}=\sum_{i=1}^{n}\left[y_{i}^{2}-10cos\left(2\pi x_{i}\right)+10\right]$$

(12) $$F_{5}=20+e-20exp\left(-0.2\sqrt{\frac{1}{n}\sum_{i=1}^{n}x_{i}^{2}}\right)-exp\left(\frac{1}{n}\sum_{i=1}^{n}cos\left(2\pi x_{i}\right)\right)$$

(13) $$F_{6}=\frac{1}{4000}\sum_{i=1}^{n}x_{i}^{2}-\Pi_{i=1}^{n}\left(\frac{x_{i}}{\sqrt{i}}+1\right).$$

**Figure 6:**
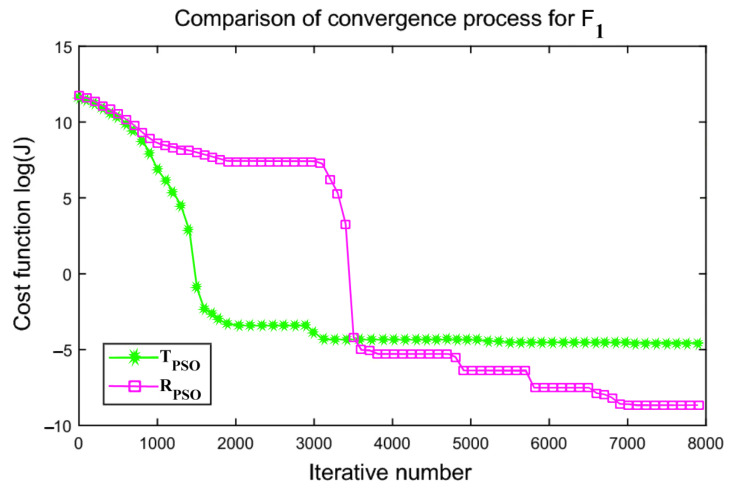
Function F_1_ comparisons for convergence process T_PSO_ and R_PSO_.

**Figure 7:**
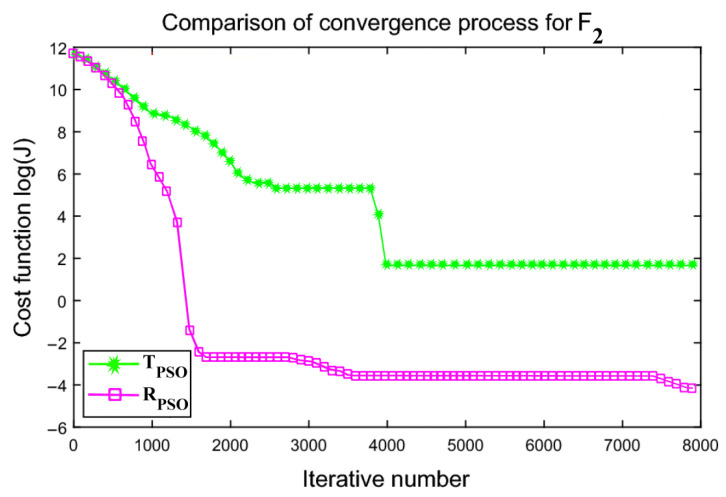
Function F_2_ comparisons for convergence process T_PSO_ and R_PSO_.

**Figure 8:**
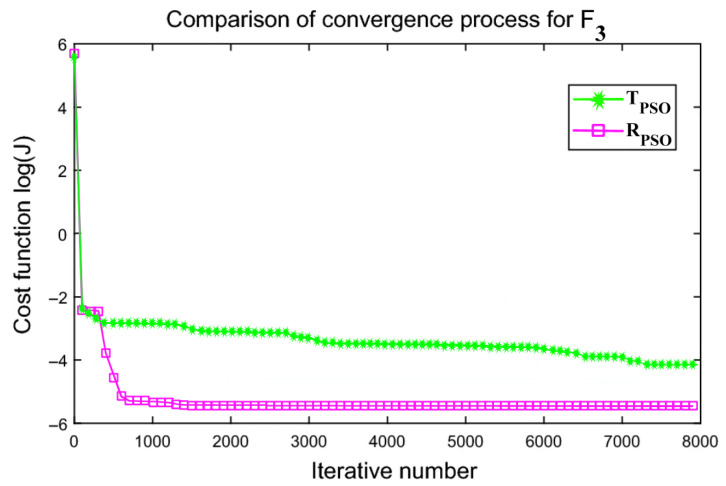
Function F_3_ comparisons for convergence process T_PSO_ and R_PSO_.

**Figure 9:**
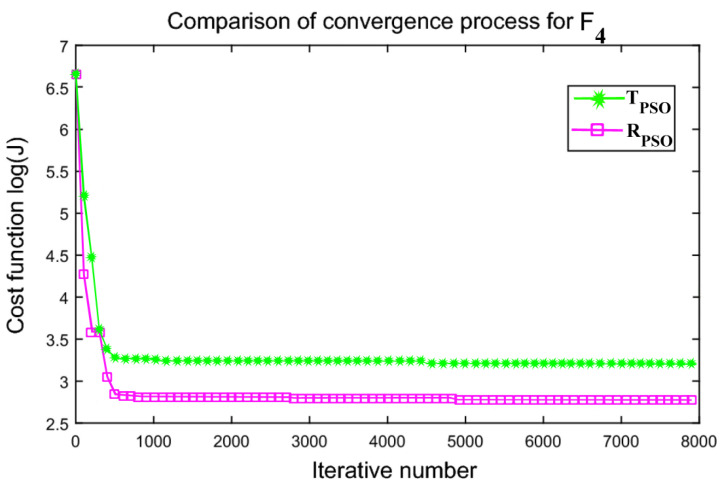
Function F_4_ comparisons for convergence process T_PSO_ and R_PSO_.

**Figure 10:**
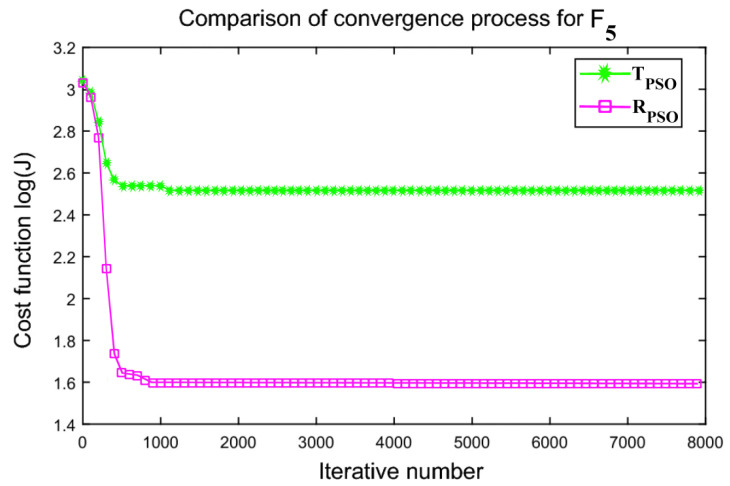
Function F_5_ comparisons for convergence process T_PSO_ and R_PSO_.

**Figure 11:**
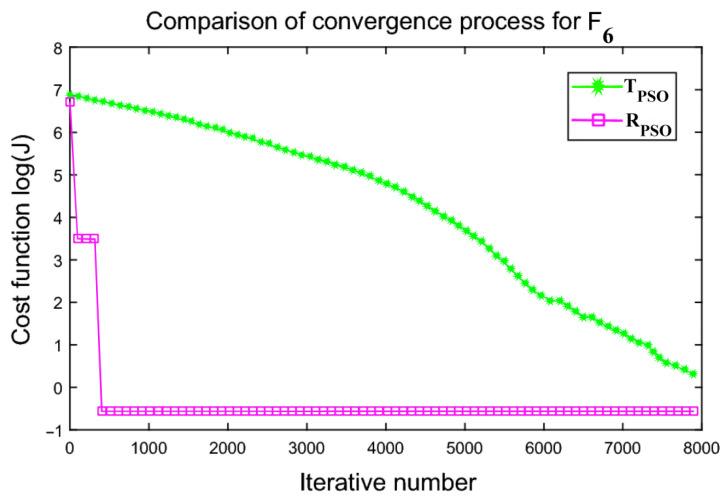
Function F_6_ comparisons for convergence process T_PSO_ and R_PSO_.

**Figure 12:**
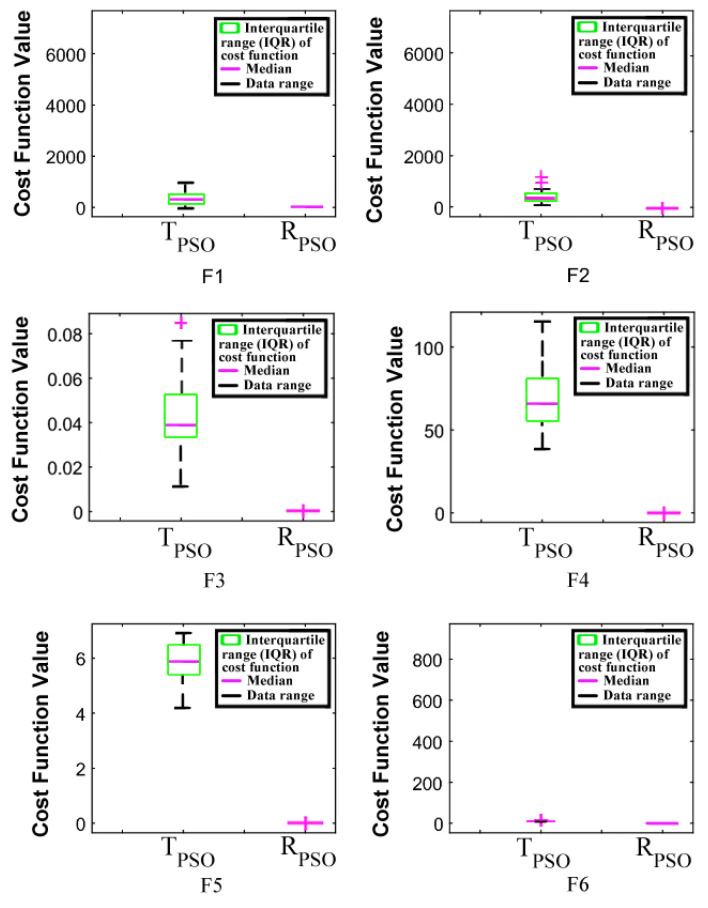
A boxplot comparison between T_PSO_ vs. R_PSO_

An interactive comparison of T_PSO_ algorithms versus R_PSO_ algorithms is illustrated in Figure 6, Figure 7, Figure 8, Figure 9, Figure 10 and Figure 11. Based on Figure 6, Figure 7, Figure 8, Figure 9, Figure 10 and Figure 11, the R_PSO_ algorithm converges significantly faster than the T_PSO_ algorithm. Furthermore, R_PSO_ has the lowest log(J) value, indicating that it performs optimization more accurately than T_PSO_. A boxplot of performance comparisons is shown in Figure 12, for benchmark functions F_1_ to F_6_. A comparison of benchmark functions T_PSO_ and R_PSO_ is presented in Table 1 and Table 2.

## 3. Results and Discussion

In this study, a proposed optimized up-conversion mixer is designed using 65 nm CMOS technology. It has been shown by experimental results that the high-linear up-conversion mixer that uses the R_PSO_-based algorithm can significantly improve key performance metrics when applied in radar applications. The mixer’s linearity, a critical factor in radar signal processing, has been notably enhanced through the application of the proposed R_PSO_ algorithm. The R_PSO_ algorithm effectively optimized the mixer’s parameters, leading to a substantial improvement in linearity.

**Figure 13:**
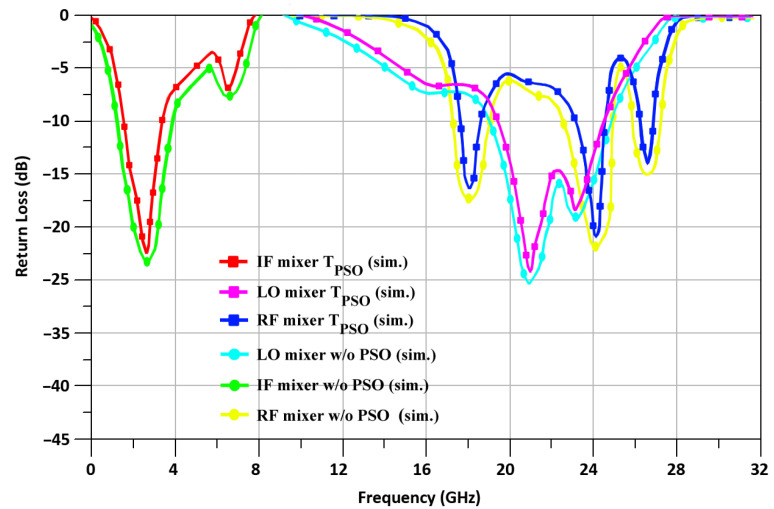
T_PSO_ and w/o PSO-based return loss of the proposed mixer.

A simulation of the return loss of T_PSO_ at RF, IF, and LO ports is shown in Figure 13. In Figure 13, we show the simulated return loss for T_PSO_ for RF, IF, and LO at −21.5, −22.1, and −24.2 dB, respectively. In addition, it shows the simulated return loss w/o PSO as −22.5, −23.8, and −25.1 at the RF, IF, and LO ports, respectively.

**Figure 14:**
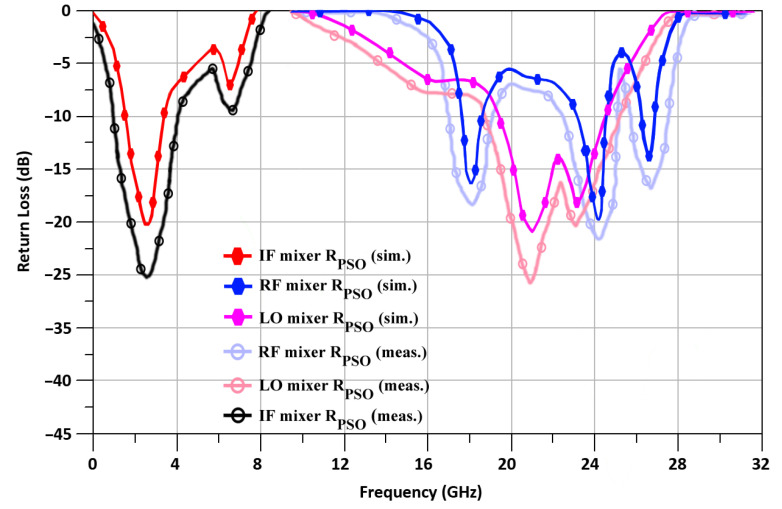
R_PSO_-based return loss of the proposed mixer.

According to Figure 14, R_PSO_ has a simulated return loss of −19.5, −20.2, and −21.1 dB, respectively, at ports RF, IF, and LO. In addition, measured return losses at the RF, IF, and LO ports, resulting in −23.8, −25.2, and −26.1, respectively.

**Figure 15:**
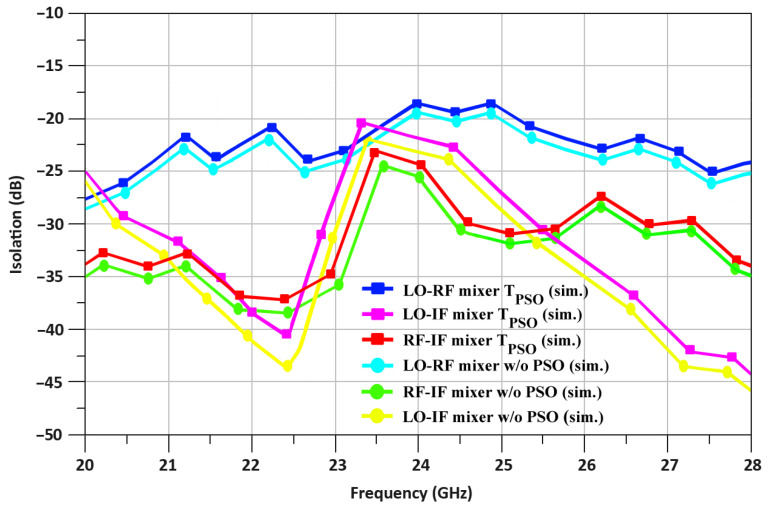
Mixer’s port isolation for T_PSO_ and w/o PSO.

In contrast, at 24 GHz, the isolation for T_PSO_ is equal to −18.1, −24.2, and −22.1 dB for the LO-RF port, RF-IF port, and the LO-IF port, respectively, is shown in Figure 15. As a result, the isolation between the LO-RF port, RF-IF port, and the LO-IF port without PSO is −19.2, −25.3, and −23.5 dB, respectively. Observing the results, it is evident that the mixer is performing well within the operating frequencies.

**Figure 16:**
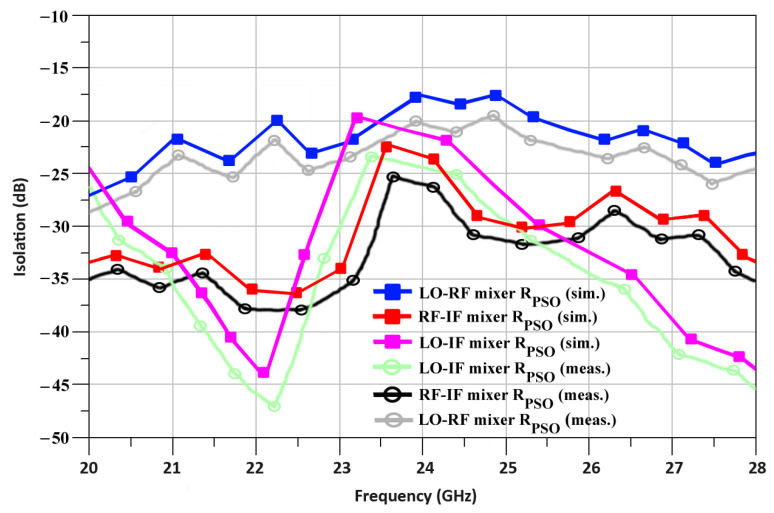
R_PSO_-based mixer’s ports isolation.

In the case of the R_PSO_, the simulated isolation between the LO-RF port, the RF-IF port, and the LO-IF port are equal to −17.1, −23.5, and −21.1 dB, respectively, at 24 GHz as shown in Figure 16. Additionally, the isolation for the R_PSO_, measured between the LO-RF port, the RF-IF port, and the LO-IF port, is equal to −20.3, −26.1, and −24.5 dB, respectively.

**Figure 17:**
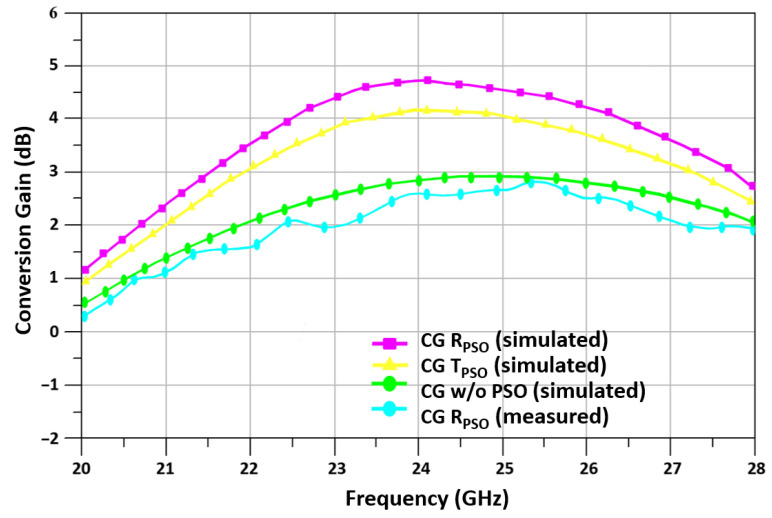
R_PSO_-based mixer CG vs. RF frequency.

For a 24 GHz-optimized up-conversion mixer, Figure 17 shows the simulated and measured result of conversion gain (CG) versus frequency. According to measured results, the proposed mixer achieves 2.5 dB CG, whereas the simulated mixer achieves 4.67 dB.

**Figure 18:**
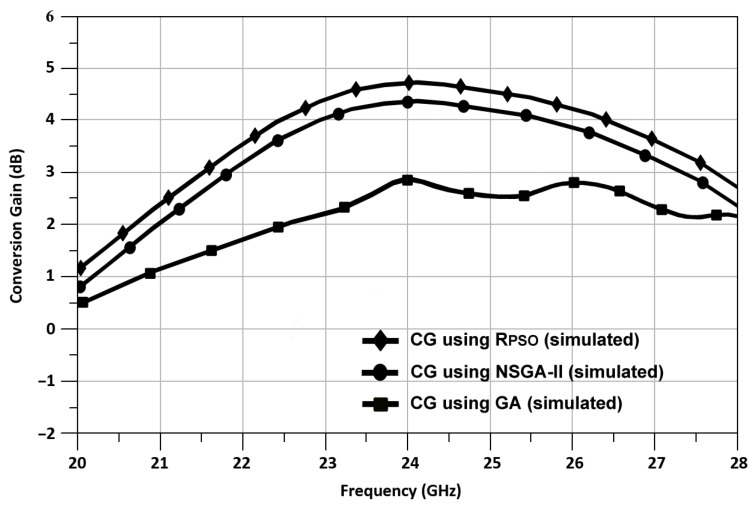
Mixer CG vs. RF frequency using R_PSO_, NSGA-II, and GA.

As shown in Figure 18, the simulated result of conversion gain (CG) versus frequency as compared with the results of another optimization algorithm such as NSGA-II, GA is shown in Figure 18. According to the simulation results, the CG of the mixer using R_PSO_ achieved 4.67 dB, while NSGA-II and GA achieved 4.2 dB and 2.81 dB, respectively.

**Figure 19:**
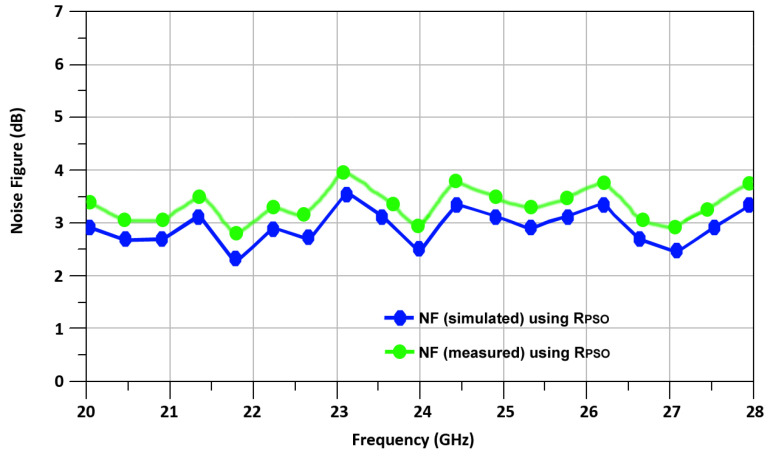
R_PSO_ mixer NF vs. RF frequency.

Figure 19 shows the R_PSO_-based mixer NF vs. RF frequency. The measured NF of the optimized up-conversion mixer is 3.1 dB.

**Figure 20:**
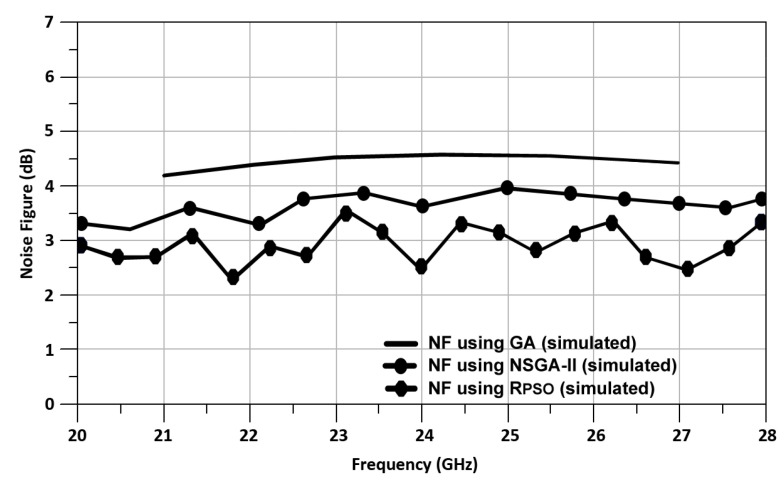
Mixer NF vs. RF frequency using R_PSO_, NSGA-II, and GA.

Figure 20 shows the mixer NF vs. RF frequency using R_PSO_, NSGA-II, and GA. The simulated NF of the optimized up-conversion mixer using R_PSO_, NSGA-II, and GA is 2.4 dB, 3.7 dB, and 4.6 dB, respectively.

**Figure 21:**
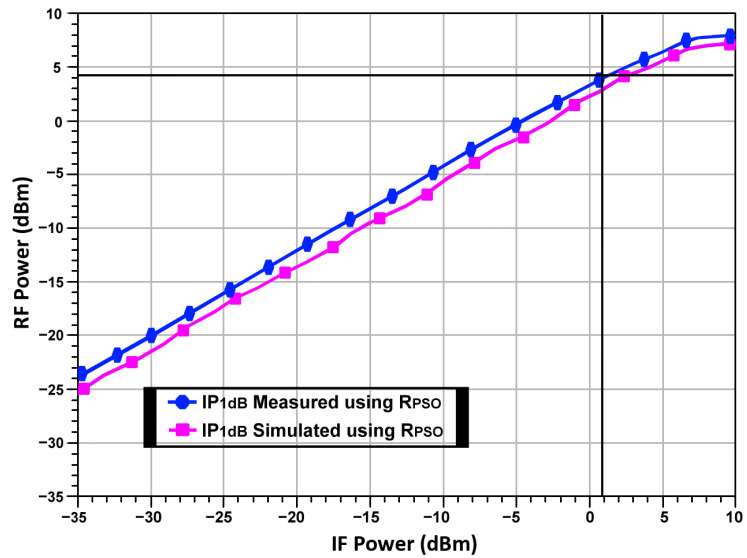
R_PSO_-based mixer RF output power vs. IF input power.

Linearity is a crucial parameter in radar applications as it directly impacts the accuracy and reliability of the received signals, especially in the presence of strong interference. The study utilized a R_PSO_-based optimization technique to enhance the linearity of the up-conversion mixer. Figure 21 depicts the R_PSO_-based mixer RF output power vs. the IF input power. The OP_1_dB of the optimized mixer is 4.2 dBm. The results indicate a significant improvement in linearity. Table 3 shows the performance comparison of the R_PSO_-based optimized up-conversion mixer with the prior research.

### Time Complexity

**Figure 22:**
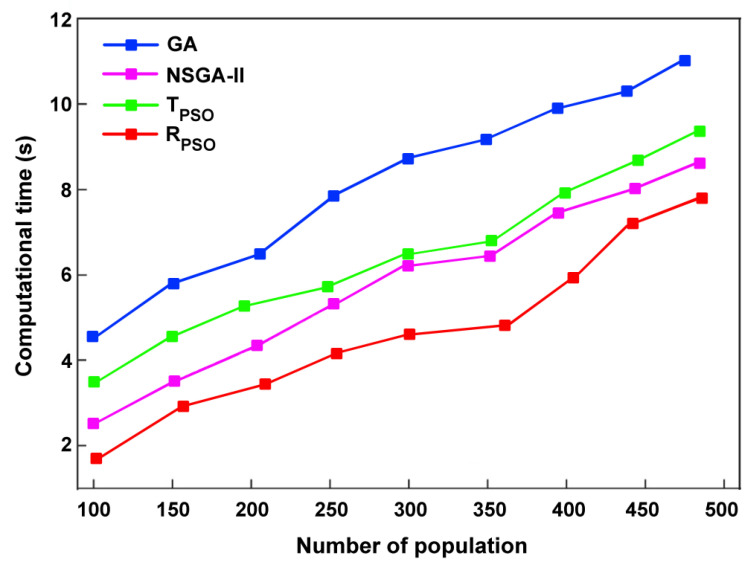
Time complexity analysis using T_PSO_, R_PSO_, NSGA-II, and GA.

In optimization algorithms, time complexity is one of the most significant parameters. It measures how quickly an algorithm solves a problem as a function of the input size. Figure 22 shows the time complexity analysis using T_PSO_, R_PSO_, NSGA-II, and GA. Our work shows that R_PSO_ has a lower time complexity than traditional GA, since particles traverse the solution space. As opposed to GAs, which work by crossovers and mutations, GAs tend to be more time-complex, especially when large populations are involved. Also, NSGA-II, designed specifically for multi-objective optimization and its time complexity, is influenced by the number of objectives and the size of the population. It takes an average of 4.535 s for R_PSO_ to design a up-conversion mixer. According to T_PSO_, it is 6.527 s, for NSGA-II it is 6.315 s, and for GA it is 8.785 s.

## 4. Conclusions and Future Research

In conclusion, this study focused on developing an R_PSO_-based high linear optimized up-conversion mixer for radar applications. The proposed mixer design demonstrated significant improvements in terms of linearity and overall performance. The optimized mixer’s OP_1_dB and CG are 4.2 dBm and 2.5 dB, respectively. Furthermore, the measured isolation between the LO-RF port, the RF-IF port, and the LO-IF port for the R_PSO_ is −20.3, −26.1, and −24.5 dB, respectively. The return losses at the three ports—the RF port, the IF port, and the LO port—were measured as −23.8 dB, −25.2 dB, and −26.1dB, respectively. The proposed R_PSO_ algorithm has been evaluated in terms of performance by comparing with other optimization algorithms such as T_PSO_ and GA, as well as NSGA-II. Simulated results indicate that R_PSO_ mixers achieve a CG of 4.67 dB while NSGA-II mixers attain 4.2 dB and GA mixers reach 2.81 dB. Also, Simulated NFs of optimized up-conversion mixers based on R_PSO_, NSGA-II, and GA are 2.4, 3.7, and 4.6, respectively. In addition, we explained and presented the result of the time complexity. In accordance with the R_PSO_ and T_PSO_, the computational time is 4.535 s and 6.527 s, for the NSGA-II it is 6.315 s, and for GA it is 8.785 s.

This study has a limitation in that the effectiveness of the R_PSO_ algorithm heavily depends on the specific characteristics of the up-conversion mixer as well as the operating conditions of the mixer, so it has the potential to be less versatile for different types of mixers. Future work may focus on further refining the optimization process, exploring additional parameters, and addressing practical implementation challenges for seamless integration into radar systems. Also, develop and explore strategies for reducing noise in the mixer, aiming to further enhance the signal-to-noise ratio. In addition, we could also explore the possibility of multi-objective optimization, where the optimization considers multiple conflicting objectives simultaneously.

## Figures and Tables

**Table 1:** T_PSO_ results of benchmark functions.

Name of the Function	F_1_	F_2_	F_3_	F_4_	F_5_	F_6_
Algorithm	T_PSO_	T_PSO_	T_PSO_	T_PSO_	T_PSO_	T_PSO_
Maximum Value	$1\times 10^{4}$	$1.01\times 10^{4}$	$6.7\times 10^{-2}$	$1.1\times 10^{1}$	$5.3\times 10^{1}$	$1.2\times 10^{1}$
Median Value	$1.03\times 10^{-2}$	$1.01\times 10^{4}$	$2.2\times 10^{-2}$	$7.7\times 10^{-4}$	4.1	$3.3\times 10^{1}$
Mean Value	$7.00\times 10^{3}$	$6.3\times 10^{3}$	$2.79\times 10^{-2}$	$3.2\times 10^{1}$	$1.2\times 10^{1}$	4.6
Minimum Value	$5.2\times 10^{-3}$	$2.02\times 10^{-5}$	$6.73\times 10^{-3}$	$1.7\times 10^{1}$	$1.1\times 10^{1}$	1.2
Standard deviation	$4.6\times 10^{3}$	$4.9\times 10^{3}$	$1.3\times 10^{-2}$	7.9	$2.2\times 10^{-1}$	2.4

**Table 2:** R_PSO_ results of benchmark functions.

Name of the Function	F_1_	F_2_	F_3_	F_4_	F_5_	F_6_
Algorithm	R_PSO_	R_PSO_	R_PSO_	R_PSO_	R_PSO_	R_PSO_
Maximum Value	$1\times 10^{4}$	$1.01\times 10^{4}$	$6.7\times 10^{-2}$	$1.1\times 10^{1}$	$1\times 10^{4}$	$1.2\times 10^{1}$
Median Value	$1.03\times 10^{-2}$	$1.01\times 10^{4}$	$2.2\times 10^{-2}$	$7.7\times 10^{-4}$	4.1	$3.3\times 10^{1}$
Mean Value	$1.1\times 10^{-2}$	$1.4\times 10^{-2}$	$2.7\times 10^{-3}$	$2.6\times 10^{1}$	4.9	8.9
Minimum Value	$2.6\times 10^{-3}$	$4.4\times 10^{-3}$	$4.9\times 10^{-5}$	$1.6\times 10^{1}$	3.5	$5.2\times 10^{-2}$
Standard deviation	$7.8\times 10^{-2}$	$6.2\times 10^{-3}$	$3.2\times 10^{-3}$	$1.4\times 10^{1}$	6.2	1.8

**Table 3:** Performance comparison of R_PSO_-based optimized up-conversion mixer.

Parameters	This Work	[22]	[23]	[24]	[25]	[26]	[27]	[28]
Technology	65	65	130	90	65	180	65	130
Freq. [GHz]	24	27.5–43.5	23.4–29.2	17.5–22.3	60	19–31	24	17
Gain [dB]	2.5	−5	−1.9	−13	−6.5	−0.8	1	−4
P_DC_ [mW]	3.24	14	38	0.149	29	38	8	93
OP_1_dB [dBm]	4.2	0.42	0.3	−0.62	−5	−8.6	−11	4.2
Chip area [mm^2^]	0.42	0.686	0.8	N/A	0.27	1	0.52	0.5

## Data Availability

Data are contained within the article.
